# qEEG spectral peak in Alzheimer’s disease: A possible tool for treatment follow-up

**DOI:** 10.1590/S1980-57642009DN20100003

**Published:** 2008

**Authors:** Irina Raicher, Daniel Yasumasa Takahashi, Paulo Afonso Medeiros Kanda, Ricardo Nitrini, Renato Anghinah

**Affiliations:** Behavioral and Cognitive Neurology Unit, University of São Paulo School of Medicine, São Paulo, Brazil.

**Keywords:** quantitative electroencephalography, spectral analysis, dementia, Alzheimer’s disease, memory complaint, cognition, qEEG, eletroencefalografia quantitative, análise espectral, demência, doença de Alzheimer, queixa de memória, cognição, EEGq

## Abstract

qEEG spectral analysis has been considered highly sensitive to cortical functional changes and agrees strongly with the clinical diagnosis of AD. The sensitivity of spectral analysis has ranged from 71% to 81% in several studies.^1-3^.

**Objective:**

The aim of this study was to retrospectively evaluate whether alpha qEEG spectral peak can supplement clinical examination by constituting an independent tool to monitor treatment and follow-up of dementia progression in Alzheimer’s disease (AD). In addition, we examined the demographic data and alpha power spectra distribution of patients and elderly normal controls.

**Methods:**

qEEGs were selected from 2 groups of patients: normal controls (n=30), and patients who fulfilled criteria for mild probable AD diagnosis (n=41). The alpha qEEG spectral analysis and MMSE were performed once or twice a year.

**Results:**

In our groups, MMSE scores and qEEG alpha spectral peak were unchanged (no statistical differences) after anticholinesterase use where qEEG spectral peak was never lower than 8 Hz in the control group.

**Conclusion:**

This study supports two important concepts. First, 8 Hz alpha appears to be the lowest awake spectral peak compatible with normality. And finally, in a clinical context, qEEG is a valuable diagnostic tool that could prove useful for Dementia follow-up.

## Abstract (pt)

A análise espectral do EEG tem sido considerada muito sensível para mudanças da função cortical e compatível com o diagnóstico clínico da Doença de Alzheimer (DA). A sensibilidade da análise espectral varia de 71% a 81% em alguns estudos.^1-3^

**Objetivo:**

A proposta deste estudo foi uma avaliação retrospectiva sobre a possibilidade do pico espectral do alfa no EEG quantitativo (EEGq) complementar o exame clínico fornecendo uma ferramenta independente para monitorizar o tratamento e para seguimento da progressão da demência na DA. Além disso, foram examinados dados demográficos e a distribuição do espectro da potência alfa de pacientes e controles da mesma idade.

**Métodos:**

EEGq foram selecionados de dois grupos de pacientes: os controles sem alterações e queixas de memória (n=30) e os pacientes com critérios preenchidos para DA provável (n=41). A análise do pico espectral do alfa no EEGq e o Mini-Exame do Estado Mental foram realizados uma ou duas vezes no ano.

**Resultados:**

Neste grupo estudado, o Mini-Exame do Estado Mental e o pico espectral do alfa no EEGq não foram estatisticamente diferentes depois do uso de anticolinesterásicos e o pico espectral do EEGq não foi menor do que 8 Hz em todos indivíduos do grupo controle.

**Conclusão:**

Este estudo sustenta dois conceitos importantes. Primeiro, 8 Hz de pico espectral de alfa, em vigília, aparenta ser o menor valor compatível com normalidade. E finalmente, em contexto clínico, EEGq é uma ferramenta que pode ser útil no seguimento da de Demência.

As the aging population continues to grow at a vigorous pace, the prevalence of neurodegenerative diseases is rising worldwide. Alzheimer’s disease (AD) is the most common neurodegenerative disorder and its treatment requires early detection of cognitive decline. Nevertheless, there is no consensus on methods to estimate and measure the diagnosis and progression of patients with AD. The advance in quantitative electroencephalography (qEEG) plays a significant role in EEG-based clinical diagnosis and studies of brain function, revealing new possibilities for clinical application in cognitive and behavioral areas. Quantitative EEG techniques include the mathematical transformation of brain electrical activity data from the time domain into the frequency domain by the Fast Fourier Transform algorithm. Traditional EEG methods support the separation of the frequency data into four main frequency bands: delta (1–4 Hz), theta (4–8 Hz), alpha (8–13 Hz), and beta (13–30 Hz) (Kaplan and Sadock, 1998). According to a longitudinal study, the mean posterior dominant frequency declined by 0.08 Hz per year of age over 60 years (Wang and Busse, 1969). There is thus ample evidence in the literature to consider an average alpha frequency of less than 8.5 Hz as abnormal, measured with the patient fully alert (Niedermeyer, E). Although posterior frequency decline is usually inespecific and cannot differentiate any particular disorder, it has been a common electroencephalographic sign described in many conditions evolving to cognitive alterations, and is frequently encountered in AD individuals. There is a good correlation between the degree of EEG abnormality and cognitive impairment in the literature. We proposed that qEEG spectral peak could represent an independent tool to perform Alzheimer’s disease follow-up after medication.

## Objective

The aim of the study was to evaluate whether the qEEG can supplement the clinical examination by constituting an independent tool to monitor treatment and dementia progression. We examined the distribution of qEEG alpha spectral peak in patients with Alzheimer’s disease (AD) and elderly normal controls, along with the impact of acetylcholinesterase inhibitor (AChEI) treatment on background qEEG activity.

## Methods

### Participants

In a retrospective study, three hundred medical files of patients older than 60 years, from a private Neurology outpatient unit, were studied. Two groups of patients were selected with the following criteria: Group 1, cognitively healthy patients with no sustained memory loss complaints and normal routine activities were considered controls (n=30); Group 2, patients who fulfilled criteria for mild and moderate probable AD diagnosis (n=41). A diagnosis of probable AD was made by an experienced consultant neurologist according to the NINCDS-ADRDA criteria.^4^ Patients presented mild to moderate symptoms, according to the DSM-III-R.^5^ Patients and controls were submitted to the Brazilian version of the Mini-Mental State Examination (MMSE)^6,7^, considering subsequent scores for the same individual as evaluation of cognitive status. All patients and controls were screened for concomitant neurologic diagnoses and psychiatric history.

## Materials and procedure

One hundred seventy qEEGs were retrieved from the files. They had been recorded during awake, resting conditions with eyes closed, by means of a computer-based system (EMSA) from 20 electrode locations using a standard International 10-20 electrodes system^8^. The EEG was bandpass filtered for 1-30 Hz prior to digitalization, using a sampling rate of 200 Hz. Samples were selected by visual inspection, in order to obtain a minimum of 20 2-s epochs free of eye blink, drowsiness, muscle movements, or equipment-related artifacts. Frequency domain analysis was performed using the Fast Fourier Transform algorithm, with the calculation of the mean frequency and average electrical power in each frequency band of the spectrum. Power was divided into the four frequency bands delta (0.5–3.9 Hz), theta (4–7.9 Hz), alpha (8–13 Hz), and beta (14.5–30 Hz). The spectral analysis of further qEEG and the MMSE tests were performed once or twice a year in the control group and in AD patients before and after 6 months of acetylcholinesterase inhibitor (AChEl) treatment. The medications used were galantamine (21 patients), memantine plus rivastigmine (1 patient), tacrine (2 patients), and rivastigmine (16 patients), donepezil (1 patient) and were grouped altogether.

### Data analysis

The Chi-square test was used to assess comparisons of sex and schooling. The two-sample *t* test was used to assess comparisons of age and the non-parametric Mann-Whitney test for awake qEEG prior to and during treatment. All statistical analyses were performed by the SPSS program.

## Results

### Demographic data

There were no significant differences between the groups in terms of sex, age and schooling (Table 1).

**Table 1:** Features of 71 patients grouped according to diagnosis.

	Controls (group 1) N=30	AD (group 2) N=41	p-value
**Age (years)**	72.00 ± 7.36	77.20 ± 8.08	0.070*
**Gender N (%)** Male Female	9 (30%) 21 (70%)	14 (34.15%) 27 (65.85%)	0.910**
**Schooling N (%)** Less than 8 years More than 8 years	4 (13.79%) 25 (86.21%)	14 (34.15%) 27 (68.85%)	0.100**

* Two-sample t test;

** Chi-square test.

Table 2 summarizes the baseline characteristics of the alpha spectral peak for group 1 vs. group 2 (Mann-Whitney test).

**Table 2:** Mann-Whitney test for alpha spectral peak EEG (group 1 vs group 2).

	N	Mean	StDev	Mann-Whitney U	p-value
Group 1	30	9.387	0.674	318.5	**0.001**
Group 2	41	8.34	1.38		

**Graph 1:**
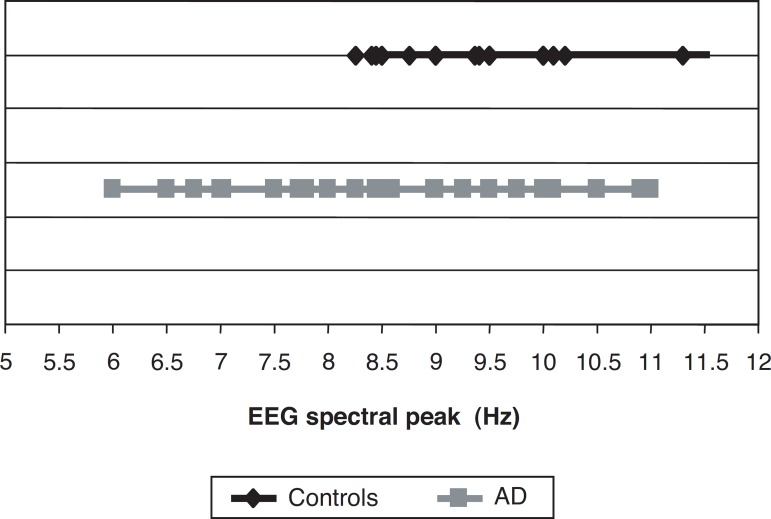
Dispersion of EEG spectral peak in this sample.

These results revealed that no control subjects had alpha spectral peak lower than 8 Hz as depicted in Graph 1.

A subgroup of 22 patients with AD diagnosis had spectral analysis of qEEG and Mini Mental State Examination (MMSE) performed twice, with a year interval before treatment and after anticholinesterase use. The results of the non-parametric Mann-Whitney test are shown in Table 3.

**Table 3:** Mann-Whitney test for cognitive performance and EEG spectral peak before and while undergoing AChEI treatment.

	Before treatment	Undergoing treatment	Mann-Whitney U	p-value
MMSE Mean (SD)	23.14 (3.34)	22.73 (4.01)	262.0	**0.956**
EEG spectral peak Mean (SD)	8.64 Hz (1.37)	8.66 Hz (1.23)	241.5	**0.991**

There was no statistically significant difference in MMSE and alpha spectral peak before and after anticholinesterase use.

## Discussion

An important finding in this study was that none of the controls had spectral peak lower than 8 Hz (Graph 1). This points to 8 Hz as one milestone of normality where all values below this on qEEGs were from AD patients. Previous research has shown that low-cost computerized qEEG techniques are able to statistically predict MCI to Alzheimer’s disease conversion,^12-14^ and could be used for prognostic purposes in early stages^15^ and during monitoring of AD treatment.^16-18^

This study investigated mainly the alpha qEEG spectral peak pattern - before and after cholinesterase inhibitor treatment. The qEEG values, performed 6 months and 1 year after medication, agreed with AD patient MMSE scores. All patients selected had good response to medica tion and their treatment was associated with no significant differences between the pre-and post-treatment alpha spectral peak (Table 3). We did not investigate whether these results were dependent on the medication used (galantamine, memantine, tacrine and rivastigmine) because our AD group was small. This limitation could be eliminated in future studies on larger samples.

## Conclusions

This study supports two important concepts. First, 8 Hz alpha appears to be the lowest awake spectral peak compatible with normality. And finally, in a clinical context, qEEG is a valuable diagnostic tool that could prove useful for Dementia follow-up. It can supplement the clinical examination by providing an independent assessment of the response to medication in AD. Alpha spectral peak analysis may quantify and measure the therapeutic response to cholinergic drugs. However, definitive validation of this procedure requires study of a larger sample of patients, already underway at our Centre.
